# Fretting Behavior of Biomimetic-Textured Silicone Rubber Under Varying Wetting Conditions

**DOI:** 10.3390/ma18163861

**Published:** 2025-08-18

**Authors:** Tengfei Zhang, Jie Su, Liaoliang Ke, Sichun Bai, Guojun Yu

**Affiliations:** 1 School of Mechanical Engineering, Tianjin University, Tianjin 300350, China; tfzhang@tju.edu.cn; 2 National Key Laboratory of Vehicle Power System, Tianjin 300350, China; 3 China North Engine Research Institute (Tianjin), Tianjin 300400, China; baisc@163.com (S.B.); yu_guojun@126.com (G.Y.)

**Keywords:** contact behavior, rubber-like material, surface texture, water/oil wetting, biomimetic tribology

## Abstract

A textured surface can significantly enhance the tribological properties of a robotic soft gripper in wet environments. However, external disturbances such as wind, sound waves, water flow, and mechanical vibrations often lead to fretting contact on the soft gripper’s surface. This article imitates the toe pad texture of tree frogs, renowned for their strong climbing abilities, to prepare silicone rubber films with hexagonal textures of different sizes and experimentally studies their fretting behavior under both deionized water and silicone oil wetting conditions. The effects of texture size, normal force, wetting condition, displacement amplitude, and frequency on the fretting behaviors of silicone rubber films are discussed in detail. The results indicate that textured surfaces significantly enhance the coefficient of friction (COF) of silicone rubber under wetting conditions with a small normal force and high frequency. Furthermore, the larger the texture size, the more noticeable the increase in COF.

## 1. Introduction

In wet environments, high friction on the surface of soft materials is a fundamental requirement for many applications, such as smart detection of wearable devices [1], crawling of intelligent robots [2], surgical graspers [3], tires [4], etc. Therefore, it is very important to enhance the tribological properties of the surface of soft materials in wet environments. To achieve better tribological performance, many scholars sought inspiration from nature [5,6,7]. It is found that surface tribological characteristics can be significantly altered through specific geometric designs [8]. Nature provides many excellent examples, including the toe pads of tree frogs [9], geckos [10], newts [11], and bush crickets [12], which exhibit outstanding friction-enhancing properties. Their toe pads feature similar polygonal micro-textures, with gaps around the textures that can expel liquid from the contact region, thus preventing slippage and generating high friction [2].

A range of polygonal textures inspired by bionics has been designed to provide high friction on surfaces of soft materials under the static friction or sliding friction condition [13]. Li et al. [14] found that the micro-dimple texture of a polydimethylsiloxane (PDMS) surface can increase friction at high sliding speeds under wetting conditions. This study mainly focused on the sliding friction at different speeds but did not investigate the friction behavior at small displacements. Hao et al. [15,16] proposed that a silicone rubber gripper with fingerprint textures can increase the actual contact area under lubrication conditions, improving gripping capability. Varenberg and Gob [12] found that hexagonal micro-textures can prevent the sliding of polyvinyl siloxane under oil wetting conditions, thereby making friction behavior stable and predictable. Xie et al. [17] observed that the wet adhesion of polyurethane elastomers with hexagonal columnar textures decreases as the aspect ratio of the channels increases. Drotlef et al. [18] prepared hydrophobic and hydrophilic microstructures on PDMS surfaces by mimicking tree frogs’ toe pads. Their results indicate that the hydrophobic surface is conducive to wet adhesion, while the hydrophilic surface is beneficial for increasing friction. Zhang et al. [3] designed double-layer textures on the PDMS surface, whose friction is approximately twice that of single-layer textures. Li et al. [19] found that the multi-layer hexagonal structure of PDMS could accelerate the rupture of the lubrication film, thereby increasing the wettability and sliding friction of the hydrophilic surface. In addition, Chen et al. [20] and Iturri et al. [21] discussed the influences of the anisotropy characteristics of hexagonal textures on the friction behavior of PDMS.

However, the practical application environments for soft materials are not always in a static or sliding state. Taking the robot soft gripper as an example, it may be affected by wind, sound waves, water flow, and mechanical vibrations during the grasping and manipulation processes, leading to fretting contact on the surface of soft materials. Fretting refers to the relative movement with very small amplitude between two contact surfaces [22]. When two objects experience fretting contact, surface damage may occur, which significantly shortens the service life of the components [23]. Currently, there are only a few studies on hard materials with surface textures under fretting conditions. Okamoto et al. [24] conducted fretting tests on bearing steel with different textured surfaces under oil lubrication. Cao et al. [25] found that an appropriate texture on the surface of titanium alloy can greatly enhance fretting performance and reduce abrasive wear. Wang et al. [26] carried out fretting tests on Ti-6Al-4V alloys with different textures and analyzed the COF curves, systematic deformation, and wear morphology in detail. There are relatively few studies on the fretting behavior of soft materials with surface textures [27,28], especially under wet conditions. In-depth research on these issues will help improve the grasping ability and stability of robot soft grippers in wet environments. Therefore, it is crucial to investigate the fretting behavior of soft materials with textures in wet environments.

In this paper, silicone rubber is chosen as the experimental material. Inspired by the tree frog’s toe pad, hexagonal textures with different sizes are prepared on the surface of silicone rubber films. The fretting behavior of the textured silicone rubber films subjected to a flat indenter is studied in deionized water and silicone oil wet environments. The fretting experiment, involving linear reciprocating motion in a flat-to-flat contact mode, is conducted. The effects of texture size, normal force, displacement amplitude, and frequency on fretting regime, friction force, and COF under water and oil wetting conditions are systematically discussed.

## 2. Materials and Methods

### 2.1. Materials of Experimental Samples and Preparation of Surface Texture

Silicone rubber is widely used in wearable devices, intelligent robots, and surgical graspers due to its ease of manufacture, low toxicity, durability, and low mechanical damping coefficient [29]. Therefore, we adopt silicone rubber as the sample material for the fretting experiment. Smooth-Sil 950 Platinum Silicone (Smooth-On Ltd., Macungie, PA, USA) is used to make silicone rubber film samples with different surface textures. It is prepared by mixing curing agent (Part A) and silicone rubber (Part B) in a ratio of 1:10, then curing at room temperature for 24 h. During the curing process, the material undergoes negligible shrinkage (shrinkage rate < 0.001). After curing, the silicone rubber sample has a hardness of 50 Shore A and a Young’s modulus of 1.87 MPa.

**Figure 1:**
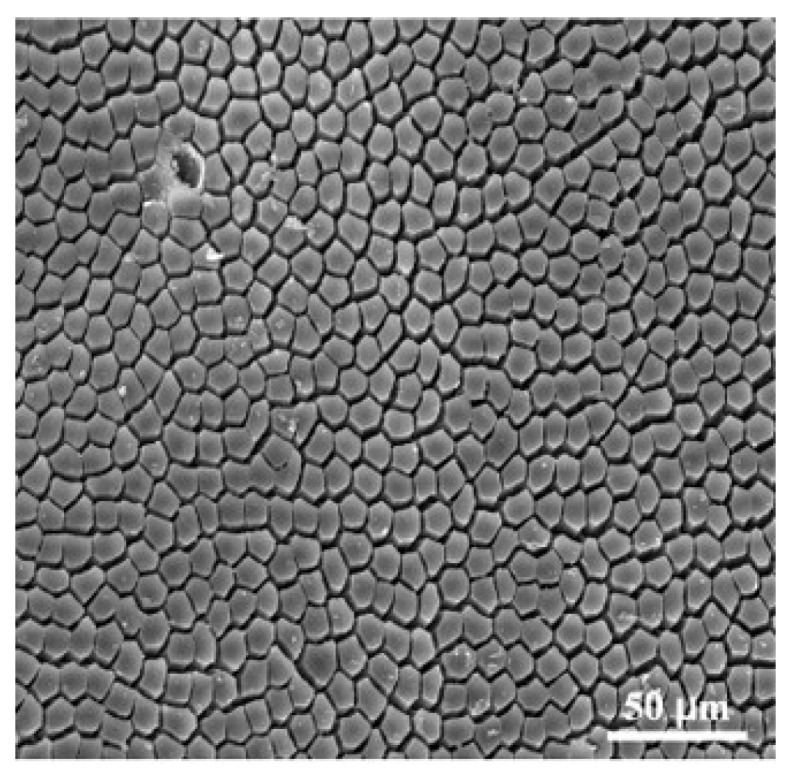
The SEM image of skin texture of a tree frog toe pad [20].

**Figure 2:**
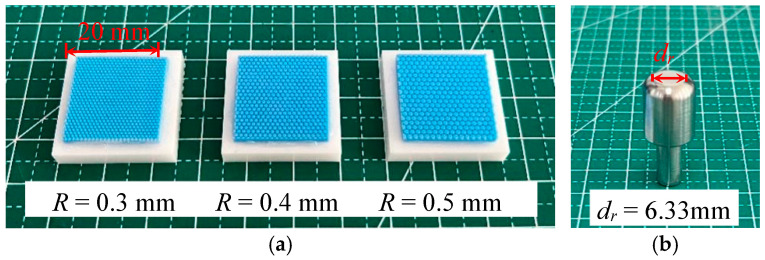
Experimental samples: (**a**) silicone rubber films with different texture sizes and (**b**) flat indenter.

Compared to other textures, the hexagonal microstructure, which resembles the structure of the toe pads of tree frogs, exhibits more stable friction [30]. The hexagonal texture may provide a larger contact surface and better adhesion under water flow impact [31,32,33]. Figure 1 shows the epithelial cell structure of the toe pad [20], where the surface is closely arranged with polygonal columns separated by channels. Inspired by this structure, hexagonal textures in Figure 2a are prepared on the surface of silicone rubber. The texture size is represented by the inner circle radius *R* of the hexagon. Three different sizes are considered: *R* = 0.3 mm, 0.4 mm, and 0.5 mm. Additionally, the height and spacing of the hexagonal texture are 0.4 mm and 0.2 mm, respectively.

The process for preparing the textured silicone rubber films is outlined as follows: (1) design the 3D models of molds with different textures using SOLIDWORKS software (2023, Dassault Systemes, Waltham, MA, USA); (2) print the resin molds using light-curing 3D printing technology (AUTOCERA, TenDimensions, Beijing, China); (3) mix silicone rubber (part A) and curing agent (part B), then pour them into the mold and cure for 24 h at room temperature; and (4) cut and clean the silicone rubber film and then bond it to the ceramic substrate. Additionally, the same procedure is used to prepare silicone rubber samples with smooth surface for comparison.

The silicone rubber sample, with a side length of 20 mm, is adhered to the ceramic substrate using an instant adhesive (Scotch-Weld CA40H, 3M, St. Paul, MN, USA) (Figure 2a). The ceramic substrate is made of zirconia (3Y-TZP) with a Young’s modulus of 200 GPa and a density of 6000 kg/m^3^. The substrate has a side length of 25 mm and a thickness of 5 mm. Figure 2b shows the stainless steel cylindrical flat indenter with a density of 8000 kg/m^3^ and a Young’s modulus of 200 GPa. The material parameters are all provided by the manufacturer. The edge of the indenter is chamfered to prevent stress concentration from affecting the accuracy of the test results. Therefore, the real diameter, *d_r_*, of the contact surface of the indenter is 6.33 mm.

The present study considers two wetting conditions: deionized water and silicone oil (Xiameter PMX-200, Dow, Midland, MI, USA). The kinematic viscosities of water and oil are approximately 1 mm^2^/s and 10 mm^2^/s at 25 °C, respectively.

### 2.2. 3D Morphology Characterization

**Figure 3:**
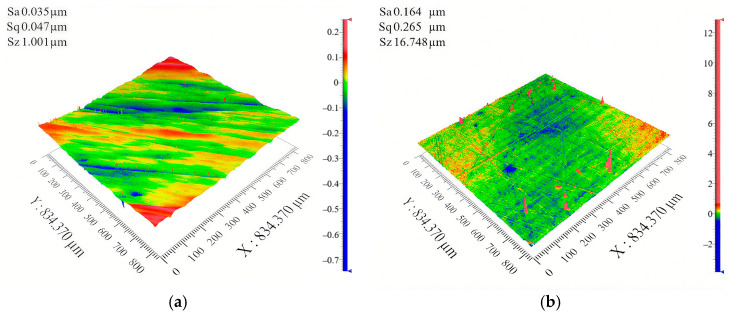
Three-dimensional morphologies of samples: (**a**) stainless steel flat indenter and (**b**) silicone rubber with a smooth surface.

**Figure 4:**
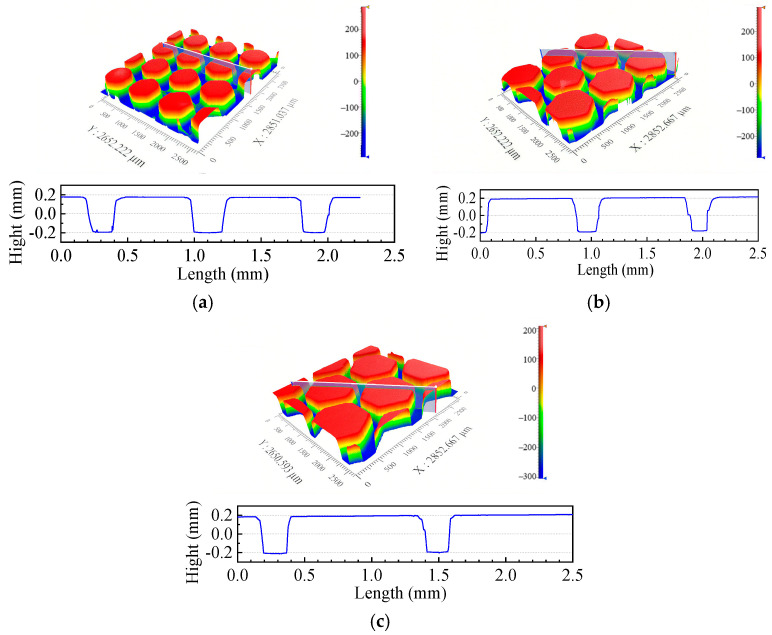
Three-dimensional morphologies of the surface of silicone rubber films with surface textures of (**a**) *R* = 0.3 mm, (**b**) *R* = 0.4 mm, and (**c**) *R* = 0.5 mm.

To evaluate whether the surface roughness and texture of samples meet the experiment requirements, the 3D morphologies of the flat indenter and the silicone rubber samples are shown in Figure 3 and Figure 4, measured using a 3D white-light interfering profilometer (Zegage^TM^ Plus, Zygo, Middlefield, CT, USA). The flat indenter and the non-textured silicone rubber have surface roughness, *S_a_*, of 0.035 μm and 0.164 μm, respectively (Figure 3). In Figure 3, the value 834.870 µm represents the measurement field of view. The resolution of the profilometer is 0.001 µm, and since no further systematic error is available, the uncertainty interval is estimated as ±0.001 µm. Compared to the surface texture height of 0.4 mm, the surface roughness has a negligible impact on fretting behavior. Figure 4 gives the height profile curves of different textures at the position of the white line. It is observed that the inner circle radius, texture spacing, and texture height of the hexagonal texture meet the design requirements.

### 2.3. Fretting Test

**Figure 5:**
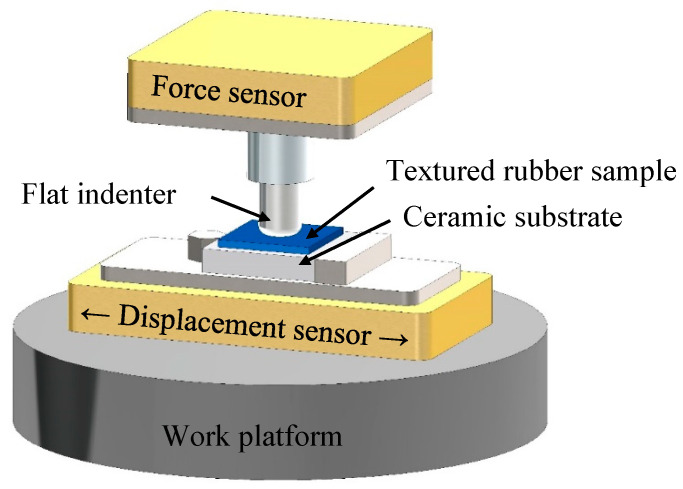
Schematic of fretting wear tester.

The fretting tests are performed using a multifunctional friction tester (UMT-TriboLab, Bruker, Billerica, MA, USA). The high-speed linear reciprocating module is selected for the fretting test in a flat-to-flat contact configuration. The stainless-steel flat indenter is fixed to the upper assembly with the help of a clamp (as shown in Figure 5). This upper assembly is fixed in the horizontal direction and can only move vertically. The force sensor in the upper assembly measures the normal and tangential forces during the testing process, with an accuracy of 0.01 N. The silicone rubber film sample is clamped to the lower assembly using an eccentric screw. The high-torque motor drives the lower assembly to achieve reciprocating motion, with an adjustable stroke range of 0.1 to 25 mm.

To capture various fretting states (i.e., partial slip, mixed fretting, and gross slip) [22], the ranges of the test parameters are as follows: normal force, *F_n_*, from 2 N to 15 N; frequency, *f*, from 2 Hz to 20 Hz; and displacement amplitude, *d*, from ±0.2 mm to ±1.2 mm. Additionally, the number of fretting cycles is *N* = 10,000.

Before the test, the silicone rubber sample and steel indenter are ultrasonically cleaned in deionized water and anhydrous ethanol, respectively, and allowed to dry for 24 h. After securing the sample on the tester, 0.05 mL of water or oil is dropped onto the surface of the sample to create a wet environment. The required normal force is then applied and held for 5 s to ensure stable contact between the friction pairs. The ambient temperature and humidity are maintained at about 26 °C and 40%, respectively. Each fretting test is performed three times, and the average value is calculated.

Note that we used a fixed volume (0.05 mL) of lubricant in our experiments and intentionally allowed its natural change during the long-term fretting process—such as the gradual evaporation of water—to reflect the non-steady wetting conditions commonly encountered in practical applications. Such changes are expected to influence both the COF and the fretting regime. In future studies, we plan to systematically investigate the effect of lubricant volume on the fretting behavior.

**Figure 6:**
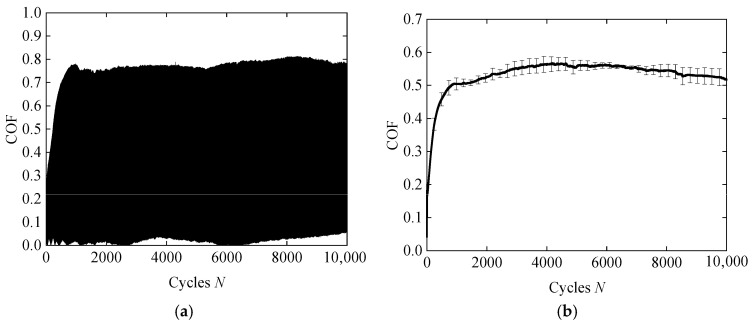
The COF of silicone rubber under *F_n_* = 5 N, *d* = ± 0.12 mm, *f* = 10 Hz, and *R* = 0 mm: (**a**) the original COF and (**b**) smoothed COF (the error bars represent the standard deviation of the three repeated tests under the same test conditions).

During the test, at least 50 data points are collected in each fretting cycle. The tester records the normal force, *F_n_*; tangential force, *F_t_*; and reciprocating tangential displacement, *D,* in real-time and automatically calculates the COF as *F_t_*/*F_n_*. The subsequent data processing is carried out using MATLAB (2023a, MathWorks, Natick, MA, USA), with the Savitzky–Golay filter applied to smooth the COF curve [34]. Figure 6a illustrates the variation in the raw COF of silicone rubber as a function of the number of cycles. Each testing condition was repeated three times to obtain averaged data. Figure 6b presents the COF results after applying the Savitzky–Golay smoothing technique [34], along with the corresponding standard deviations from the three tests.

The experimental design was primarily based on the standard test method for damage to contacting solid surfaces under fretting conditions (ASTM G204-21 [35]), as well as research studies reported in the literature [36,37].

## 3. Results and Discussion

### 3.1. F_t_-D Curves

The *F_t_-D* curve reflects the change in friction force (i.e., tangential force), *F_t_*, with tangential displacement, *D,* during the test. It also indicates the fretting regime. The *F_t_-D* curve of linear or elliptical shapes suggests that fretting contact is in the partial slip regime, where the contact center experiences adhesion and the contact edge undergoes sliding. The parallelogram-shaped curve indicates that the fretting is in the gross slip regime, meaning the two contacting bodies have undergone complete relative sliding. The mixed fretting regime lies between the gross slip and partial slip regimes. The number of cycles, displacement amplitude, and friction force have complicated relationships in this regime. The *F_t_-D* curves of various shapes and their transformations are observed in this regime, typically remaining elliptical in the relatively stable stage.

**Figure 7:**
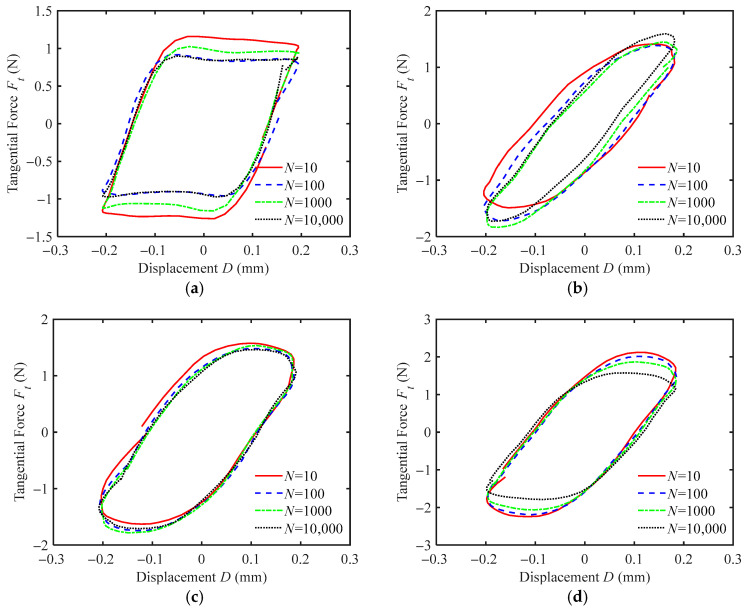
The *F_t_-D* curves of silicone rubber films with different texture sizes under water wetting condition for *F_n_* = 2 N, *d* = ± 0.2 mm, and *f* = 10 Hz: (**a**) *R* = 0 mm, (**b**) *R* = 0.3 mm, (**c**) *R* = 0.4 mm, and (**d**) *R* = 0.5 mm.

Figure 7 shows *F_t_-D* curves of silicone rubber films with different texture sizes, *R,* under water wetting conditions. At the smooth silicone rubber surface (*R* = 0 mm), *F_t_-D* curves show a parallelogram shape across different fretting cycles, indicating that there is always relative sliding between the indenter and the smooth silicone rubber surface throughout the reciprocating fretting process. This suggests that fretting is in the gross slip regime. The *F_t_-D* curves of silicone rubber films with surface textures (*R* = 0.3 mm, 0.4 mm, and 0.5 mm) are elliptical, so fretting is in the partial slip regime. This indicates that the presence of textures can effectively reduce relative sliding. The reason is that soft textures are more prone to elastic-plastic deformation than smooth surfaces, so tangential displacement is primarily coordinated by the elastic deformation of the texture itself. Therefore, the fretting remains in the partial slip regime.

Moreover, silicone rubber films with surface textures have a higher friction force, *F_t_*, than that of the smooth silicone rubber films (as shown in Figure 7). This is because the water in the contact region is drained out through the grooves of the texture, resulting in little or no water at the contact surface and thereby increasing the friction force. The fretting regime is largely unaffected by the texture size *R*. However, the maximum friction force increases as the texture size increases. This is because larger textures have a larger contact area, leading to a higher friction force.

**Figure 8:**
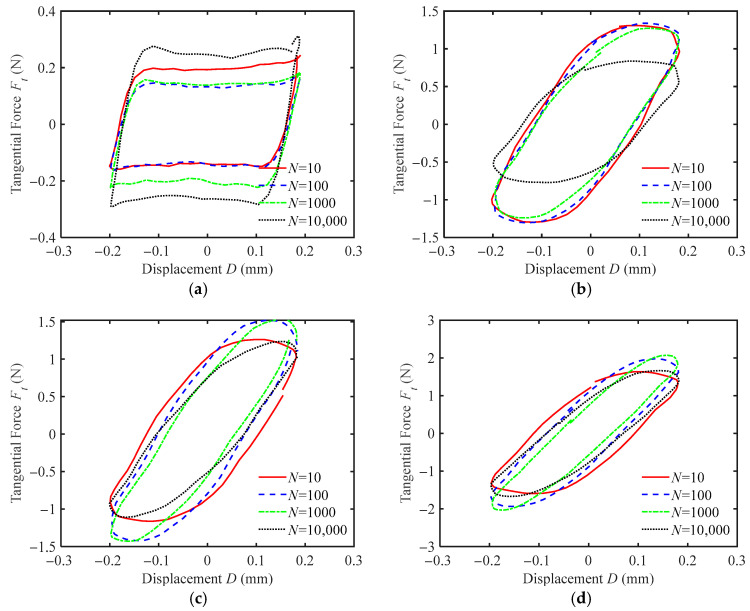
The *F_t_-D* curves of silicone rubber films with different texture sizes under oil wetting condition for *F_n_* = 2 N, *d* = ± 0.2 mm, and *f* = 10 Hz: (**a**) *R* = 0 mm, (**b**) *R* = 0.3 mm, (**c**) *R* = 0.4 mm, and (**d**) *R* = 0.5 mm.

Figure 8 shows *F_t_-D* curves of silicone rubber films with different texture sizes *R* under oil wetting conditions. The friction force on the textured surface is about five times greater than that on the smooth surface. This is because the oil can still be discharged from the contact region through the grooves, leading to two-body contact between the indenter and silicone rubber film. For silicone rubber with *R* = 0 mm, the friction force, *F_t_,* first decreases and then increases as the number of cycles grows. This behavior occurs because the oil plays a lubricating role during the initial fretting stage, reducing the friction force. As the fretting progresses, the oil is gradually pushed out of the contact region, causing a decrease in lubrication and an increase in friction. In contrast, the friction force for the textured surface first increases and then decreases as the number of fretting cycles increases. The increase in friction is due to adhesion between the two contact surfaces during the initial stage of fretting. After a certain number of cycles, the friction pair becomes fully run-in, and the wear of excess burrs and texture corners causes the friction to decrease.

By comparing Figure 7a and Figure 8a, it can be observed that under both water and oil wetting conditions, the fretting on the smooth silicone rubber surface is in a gross slip regime. However, the friction force under oil wetting conditions is significantly smaller than that under water wetting conditions, with the maximum friction force reduced by about three times. Silicone rubber is a hydrophobic and lipophilic material. Compared to water, silicone oil has a higher viscosity, better thermal stability, and is less prone to evaporation. Under identical experimental conditions, oil tends to remain at the contact interfaces, providing stable lubrication and reducing friction. For silicone rubber films with surface textures, the fretting behavior under both water and oil wetting conditions is in the partial slip regime, and the magnitude of the friction force is similar (as shown in Figure 7b–d and Figure 8b–d). The friction force increases as the texture size increases. This indicates that the wetting condition has a negligible impact on the fretting contact behavior of textured silicone rubber.

### 3.2. COF Curves

**Figure 9:**
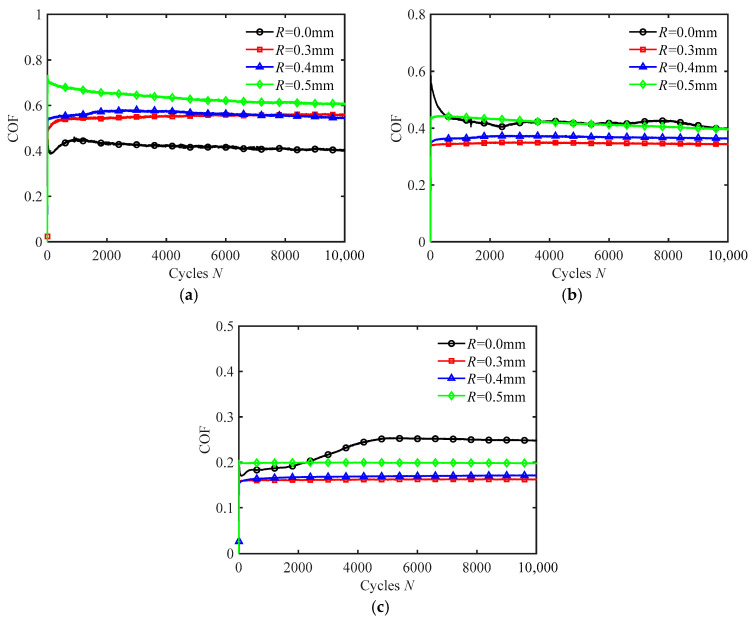
The COF curves of silicone rubber films with different texture sizes in water wetting condition for different normal forces under *d* = ± 0.2 mm and *f* = 10 Hz: (**a**) *F_n_* = 2 N, (**b**) *F_n_* = 5 N, and (**c**) *F_n_* = 15 N.

Figure 9 presents the COF curves of silicone rubber films with different texture sizes under water wetting conditions for different normal forces. As the normal force increases, the COF of the smooth surface gradually transitions from being lower than that of the textured surface to approaching it and eventually exceeds it. In other words, the textured surface can effectively increase the friction force of the silicone rubber under small normal forces. For the small normal force (*F_n_* = 2 N), the fretting of the smooth surface is in the gross slip regime, allowing water to easily enter the contact region and act as a lubricant. However, for the textured surface, the water is easily expelled through the grooves. And, in the partial slip regime, it is difficult for water to reach the contact region, minimizing the lubrication effect. As a result, the COF of the textured surface is larger than that of the smooth surface. As the normal force increases, the partial slip regime becomes more likely to occur [24]. For the large normal force (*F_n_* = 15 N), water on the smooth surface is easily squeezed out and does not re-enter the contact region under partial slip. Consequently, the actual contact area of the smooth surface is greater than that of the textured surface. So, for large normal forces, the COF of a smooth surface exceeds that of a textured surface.

Additionally, Figure 9 exhibits that the COF increases with the increase in texture size. For textured surfaces, the lubricating effect of water is minimal. So the larger the texture size, the larger the actual contact area and, therefore, the larger the COF. Furthermore, the COF decreases with the increase in normal force. The reason is that the increased load makes relative sliding between the contact surfaces more difficult. For a given displacement amplitude, the fretting is more prone to enter the partial slip regime, leading to a reduction in the COF.

**Figure 10:**
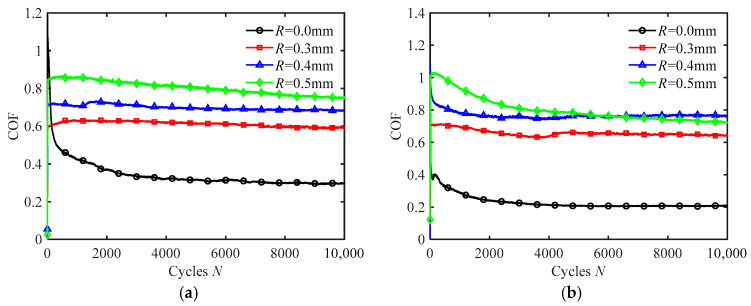
The COF curves of silicone rubber films with different texture sizes in water wetting conditions for different displacement amplitudes under *F_n_* = 2 N and *f* = 10 Hz: (**a**) *d* = ± 0.4 mm and (**b**) *d* = ± 1.2 mm.

Figure 10 shows COF curves for silicone rubber films with different texture sizes under water wetting conditions for different displacement amplitudes. The results under *d* = ±0.2 mm are given in Figure 9a. It is evident that the COF of the textured surface is consistently larger than that of the smooth surface at all displacement amplitudes. And a larger texture size corresponds to a larger COF. As displacement amplitude increases, the COF of the smooth surface tends to decrease gradually, whereas the COF of the textured surface increases. This difference is primarily attributed to the lubricating effect of water. Specifically, the increase in displacement amplitude leads to a shift in fretting regime from partial slip to gross slip [24]. For a smooth surface, water is more prone to enter the contact region under gross slip, and thus its lubricating effect reduces the COF. However, for the textured surface, the water remains trapped in the grooves and does not contribute to lubrication. As a result, for a textured surface, as displacement amplitude increases, fretting regime transitions from partial slip to gross slip, leading to an increase in COF. Overall, the larger the displacement amplitude, the greater the friction-enhancing advantage of the textured surface.

**Figure 11:**
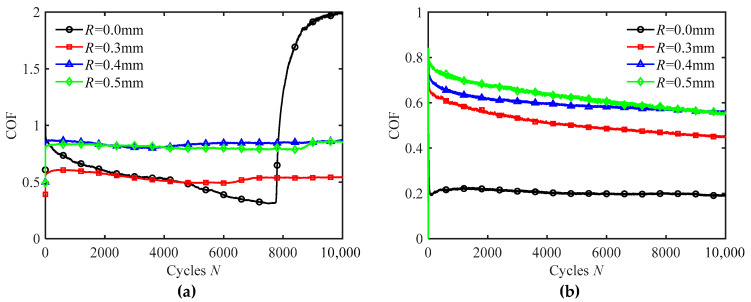
The COF curves of silicone rubber films with different texture sizes in water wetting condition for different frequencies under *F_n_* = 2 N and *d* = ± 0.4 mm: (**a**) *f* = 2 Hz and (**b**) *f* = 20 Hz.

Figure 11 plots the COF curves for silicone rubber films with different texture sizes under water wetting conditions for different frequencies. For both smooth and textured surfaces, the COF decreases with an increase in frequency. At the low frequency (*f* = 2 Hz), the textured surface maintains a stable COF throughout the fretting process. In contrast, the COF of the smooth surface decreases continuously during the initial and middle stages of fretting but begins to increase after approximately 8000 cycles, eventually exceeding the COF of the textured surface. The decrease in COF during the initial and middle stages is due to the effects of water lubrication and the mutual wear-in of the contact surfaces. In fact, the time required to complete 10,000 cycles is about 85 min at frequency *f* = 2 Hz, which is sufficient for the water on the smooth surface to be depleted during the later stages of fretting. This depletion leads to lubrication failure and an increase in the COF. At the high frequency (*f* = 20 Hz), water is more likely to form a lubrication film on the smooth surface, reducing direct contact between the indenter and the silicone rubber surface and thus maintaining a consistently low COF. However, for the textured surface, the COF decreases during fretting. This is because high-frequency fretting drives water from the grooves into the contact region, and the additional water helps to reduce friction, resulting in a decrease in COF.

**Figure 12:**
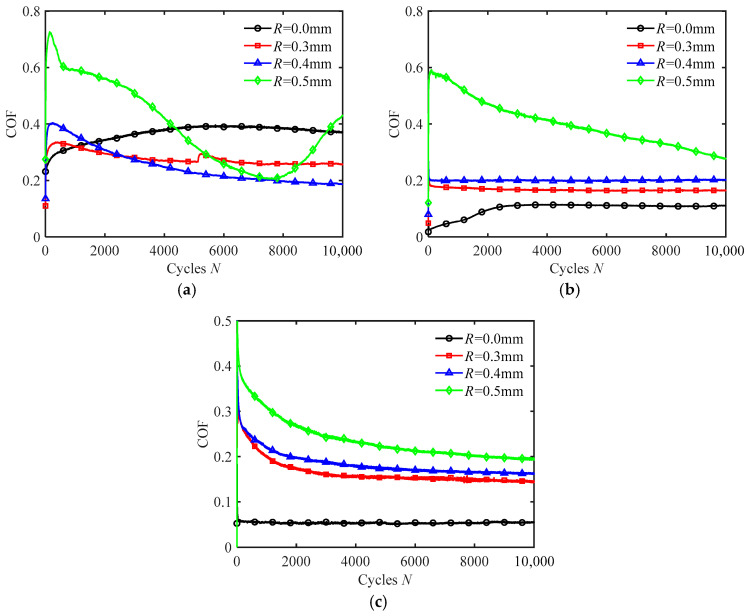
The COF curves of silicone rubber films with different texture sizes in oil wetting condition for different frequencies under *F_n_* = 2 N and *d* = ± 0.4 mm: (**a**) *f* = 2 Hz, (**b**) *f* = 10 Hz, and (**c**) *f* = 20 Hz.

Figure 12 shows COF curves for silicone rubber films with different texture sizes under oil wetting conditions for different frequencies. When *f* = 2 Hz, as the number of cycles increases, the COF of the smooth surface increases to a certain value and then stabilizes. But the textured surface does not maintain a stable high COF at low frequency. For *R* = 0.3 mm and 0.4 mm, the COF gradually decreases with the increase in the number of cycles. For *R* = 0.5 mm, the COF initially decreases and then increases. This behavior can be attributed to the reduction of oil in the contact region of the smooth surface during long-term fretting, resulting in reduced lubrication and increased COF. Silicone rubber is a hydrophobic and oleophilic material, which tends to swell in oil environments [38,39,40]. For the textured surface, the silicone rubber swells after long-term immersion in oil, reducing the groove size of the textures. This decreases the ability of the texture to expel oil, which improves lubrication and leads to a low COF. In the later stages of fretting, the grooves on the larger textures (*R* = 0.5 mm) first disappear due to swelling, leading to an increase in contact region and a reduction in oil lubrication. This results in an increase in COF. This swelling effect suggests a limitation of silicone rubber in long-term oil environments and highlights the potential need to explore alternative materials or surface treatments for improved dimensional stability. In addition, with the increase in *f*, the friction-enhancing effect of the textured surface becomes more apparent. When *f* = 10 Hz and 20 Hz, the COF of the textured surface is larger than that of the smooth surface.

Comparing Figure 11 and Figure 12, the COF under oil wetting is smaller than under water wetting. This is because silicone rubber is hydrophobic and oleophilic, meaning that silicone oil can effectively cover the contact surface and provide superior lubrication. Whether under oil or water wetting conditions, the COF consistently decreases with increasing frequency.

## 4. Conclusions

The fretting behaviors of silicone rubber films with bionic textured surfaces under a flat indenter are investigated experimentally in both deionized water and silicone oil wetting conditions. The effects of texture size, normal force, wetting condition, displacement amplitude, and frequency on the fretting behaviors of silicone rubber films are discussed. The following are the main conclusions:

(1) Texture size has little effect on the fretting regime for three different sizes: R = 0.3 mm, 0.4 mm, and 0.5 mm;
(2) For a small normal force or high frequency, the COF of the textured surface is larger than that of the smooth surface. The COF exhibits an upward trend with increasing texture dimensions;
(3) For a smooth or textured surface, the COF decreases as the normal force or frequency increases. With increasing displacement amplitude, the COF of the smooth surface decreases gradually, while the COF of the textured surface increases;
(4) At low frequency and water wetting conditions, the COF of textured surfaces remains stable throughout the fretting process, while the COF of the smooth surface first decreases and then sharply increases, eventually exceeding the COF of the textured surfaces;
(5) Under oil wetting conditions, the COF of the smooth surface remains relatively stable at low frequency, but the COF of textured surfaces first decreases and then stabilizes.

## Data Availability

The original contributions presented in this study are included in the article. Further inquiries can be directed to the corresponding authors.

## Abbreviations

Summary of abbreviation explanations:

COF	coefficient of friction
PDMS	polydimethylsiloxane
